# A Machine Learning Approach to Classifying EEG Data Collected with or without Haptic Feedback during a Simulated Drilling Task

**DOI:** 10.3390/brainsci14090894

**Published:** 2024-08-31

**Authors:** Michael S. Ramirez Campos, Heather S. McCracken, Alvaro Uribe-Quevedo, Brianna L. Grant, Paul C. Yielder, Bernadette A. Murphy

**Affiliations:** 1 Faculty of Health Sciences, Ontario Tech University, Oshawa, ON L1G 0C5, Canada; michael.ramirez@mail.escuelaing.edu.co (M.S.R.C.); heather.mccracken@ontariotechu.net (H.S.M.); brianna.grant@ontariotechu.net (B.L.G.); paul.yielder@ontariotechu.ca (P.C.Y.); 2 Department of Biomedical Engineering, University Colombian School of Engineering Julio Garavito, Bogota 111166, Colombia; 3 School of Medicine and Health Sciences, Universidad del Rosario, Bogota 111221, Colombia; 4 Faculty of Business and Information Technology, Ontario Tech University, Oshawa, ON L1G 0C5, Canada; alvaro.quevedo@ontariotechu.ca

**Keywords:** machine learning, haptic feedback, electroencephalography (EEG), simulations

## Abstract

Artificial Intelligence (AI), computer simulations, and virtual reality (VR) are increasingly becoming accessible tools that can be leveraged to implement training protocols and educational resources. Typical assessment tools related to sensory and neural processing associated with task performance in virtual environments often rely on self-reported surveys, unlike electroencephalography (EEG), which is often used to compare the effects of different types of sensory feedback (e.g., auditory, visual, and haptic) in simulation environments in an objective manner. However, it can be challenging to know which aspects of the EEG signal represent the impact of different types of sensory feedback on neural processing. Machine learning approaches offer a promising direction for identifying EEG signal features that differentiate the impact of different types of sensory feedback during simulation training. For the current study, machine learning techniques were applied to differentiate neural circuitry associated with haptic and non-haptic feedback in a simulated drilling task. Nine EEG channels were selected and analyzed, extracting different time-domain, frequency-domain, and nonlinear features, where 360 features were tested (40 features per channel). A feature selection stage identified the most relevant features, including the Hurst exponent of 13–21 Hz, kurtosis of 21–30 Hz, power spectral density of 21–30 Hz, variance of 21–30 Hz, and spectral entropy of 13–21 Hz. Using those five features, trials with haptic feedback were correctly identified from those without haptic feedback with an accuracy exceeding 90%, increasing to 99% when using 10 features. These results show promise for the future application of machine learning approaches to predict the impact of haptic feedback on neural processing during VR protocols involving drilling tasks, which can inform future applications of VR and simulation for occupational skill acquisition.

## 1. Introduction

Artificial Intelligence (AI), computer simulations, and virtual reality (VR) are increasingly being leveraged to create and implement training protocols and educational resources. VR and simulations are particularly important for high-risk occupations and other scenarios where users are exposed to safe and controlled environments that have limited opportunities for real-world settings [1,2,3,4,5,6]. VR is increasingly being used in rehabilitation settings [7,8,9], among others [10,11].

Virtual simulations can be developed to integrate a wide array of realistic multisensory feedback, including but not limited to haptic, auditory, and visual information. Haptic feedback, which is defined as the sense of touch and includes tactile, kinesthetic, and proprioceptive awareness [12], is increasingly becoming an integral component of high-fidelity simulation. Users can experience haptic sensations via vibrations, pulses, thermal changes, or rumbles [13]. Incorporating haptic feedback into motor skill acquisition paradigms has been found to enhance sensorimotor skills, particularly for skills that require force modulation for successful performance [14]. Haptic feedback is also important for the rehabilitation of individuals with neural deficits such as multiple sclerosis [8,9] and Guillain–Barré [7] or cognitive and balance deficits in elderly populations where there is greater reliance on multisensory integration due to unisensory deficits [10,11].

Developing an enhanced understanding of physiological constraints associated with VR is fundamental to informing the way in which VR training is developed and implemented. Unlike self-reported surveys, biological signals, such as those obtained using electroencephalography (EEG), show promise as an objective means of assessing sensory and neural processing associated with task performance in virtual environments [15]. However, current research that has assessed EEG recorded in VR settings has resulted in heterogeneous findings. More specifically, there is limited research indicating specific EEG markers that can reliably discriminate the impact of haptic feedback in virtual environments.

Technology is rapidly evolving and AI will be critical in the venture to enhance the fidelity of computer simulations and VR, including associated motor skill acquisition, performance, and transfer to real-world physical settings. Machine learning has utility in creating experience-based predictions, and in efficiently sorting information from diverse data sets [16]. EEG datasets can be vast, often requiring substantial time post-recording to manually analyze and categorize data, limiting its utility when real-time insights are needed. As a result of advances in computing power, machine learning approaches have been designed to assess EEG data in the time and frequency domains [17]. Such approaches can extract a great number of neural features simultaneously, allowing for a high degree of precision [17]. Machine learning approaches have been successfully applied to EEG data [18,19,20,21,22,23]. 

Much of the research that has investigated VR using EEG data and machine learning approaches has been conducted to elucidate the underlying mechanism of cybersickness while in VR [24,25]. Other works include establishing markers of excitement from EEG data while in VR [26]. When combining a deep learning approach in conjunction with a machine learning approach, a classification prediction accuracy of 78–96% using EEG spectral bands was achieved [26]. When applying deep learning to a real-time rehabilitation experiment in stroke patients, Karácsony et al. achieved an accuracy rate of 78.6% [27]. Another study used a convolutional neural network (CNN) to classify EEG data collected during virtual simulations inducing varying levels of stress [28]. Their findings suggest optimal results can be achieved when utilizing delta, theta, alpha, beta, and gamma, yielding a 96.42% accuracy rate in distinguishing stress and relaxation states [28]. These outcomes were achieved using multilayer perceptron (MLP) and support vector machine (SVM) classifiers [28].

It can be noted that machine learning applications for classifying and/or analyzing EEG signals are very broad. This has allowed machine learning tools to be included to evaluate the impact of haptic feedback on EEG signals. For instance, Alsuradi et al. (2020) collected 64-channel EEG signals from 26 subjects who interacted with a touch screen while sliding their index finger on virtual guitar strings, while visual and auditory feedback were presented and haptic feedback varied depending on the desired condition (with or without feedback). They used SVM and extreme gradient boosting (XGBoost) to classify the presence or absence of haptic feedback and obtained accuracy rates of 85% and 84%, respectively [29,30]. Similarly, members of the same research group assessed EEG signals from 19 participants who manipulated a haptic device that simulated a racket. The participants had to perform two different tasks, an active one where the user had to hit a ball with the racket using the haptic device and a passive one where the user only had to wait for the ball to fall and collide with the racket; in the latter case, the participant only pressed a button to hold the racket [31]. In both cases, haptic feedback was provided when the ball hit the racket. In this work, a CNN was used to classify the two tasks performed by the participants [31]. Different numbers of electrodes, including one, four, and six were used, obtaining mean accuracy percentages of 84.56%, 93.96%, and 95.89%, respectively [31]. Haptic feedback has also proved useful in emotion recognition exercises. The inclusion of haptic vibration patterns associated with an emotion appeared to enhance performance in the classification of EEG signals associated with different emotions, obtaining 85.46% accuracy when classifying four emotions. As shown by the authors, the accuracy percentages were higher when haptic vibration patterns were included than when they were not used [32].

The combination of EEG and machine learning is proving invaluable to improve VR applications moving forward. For example, a machine learning approach was proposed for use in conjunction with a VR and EEG protocol to distinguish important features of epilepsy. This proposal includes six standardized features to be assessed using a k-nearest neighbors (KNN) algorithm, with the goal of detecting photosensitivity in those with epilepsy, suggesting that machine learning could identify biomarkers of sensory processing from EEG data, including when in VR [33].

Current and future applications of machine learning approaches are important to elucidate the relationship of features within the EEG signal during VR protocols. For instance, having the ability to perform real-time recognition and classification of EEG data automatically will enhance the design and experience of VR-based training and learning protocols. This has the potential to harness biosignal features to enhance immersion and improve learning outcomes. Furthermore, this may allow for real-time modulation of training protocols ad hoc. Considering that humans rely on the sense of touch and that the application of haptic feedback includes domains such as medicine, entertainment, and robotics, it is clear that this is an important area of inquiry.

In diagnostic terms, this also opens the window to observe differences in brain processing of haptic information, which is relevant in multiple sclerosis [8,9] and Guillain–Barré [7], among others [10,11]. This work also has applications in rehabilitation and prevention. Simulations and VR are increasingly used for rehabilitation, but the role of haptics and proprioception is rarely addressed. 

For the current study, machine learning approaches were applied to high-density, 64-electrode EEG data collected with and without haptic feedback. The objective was to differentiate EEG signals representative of neural circuitry associated with haptic and non-haptic sensory feedback when completing a simulated drilling task. This will provide an important foundation for future machine learning applications to accurately categorize neural signals in real time. Additionally, if we can differentiate processing differences with haptic feedback using AI, it may lead to better design of rehabilitation interventions. The EEG data sets were analyzed based on the extraction of different types of time-domain, frequency-domain, and nonlinear features, where 360 features were tested (40 features from nine EEG electrodes). 

The objective of the current study was to assess the ability of machine learning approaches to identify features of EEG data related to haptic feedback. We sought to address the following research questions: (1) How effective are machine learning approaches at identifying haptic signals from EEG data? (2) What signal features and machine learning approach classifiers optimize the identification of haptic biosignals from EEG data? We hypothesize that machine learning approaches will yield high levels of accuracy when differentiating EEG signals associated with haptic feedback during simulations.

## 2. Materials and Methods

### 2.1. Ethical Approval

This study received ethical approval from the Ontario Tech University Research Ethics Board. All participants gave written informed consent before they participated in this study. This study was performed according to the principles set out by the Declaration of Helsinki for the use of humans in experimental research.

### 2.2. Paradigm and Data Collection

**Figure 1:**
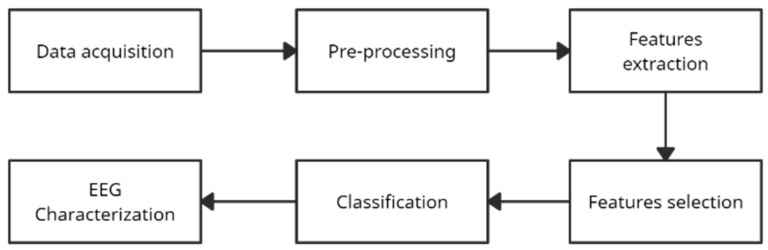
Diagram of the experimental flow for application of the machine learning algorithms to the EEG data.

The EEG recordings used were collected during a simulated drilling task at the Laboratory of Human Neurophysiology and Rehabilitation, Ontario Tech University, Oshawa, Canada (REB approval 15402). This data set contained EEG signals from 15 participants. The participants were healthy young adults, 22.9 (±1.4) years of age, who reported normal hearing and normal or corrected-to-normal vision (n = 15). Inclusion criteria required right-handedness, confirmed by the Edinburgh Handedness Questionnaire, and prior experience using a drill. The data acquisition, analysis, and characterization sequence followed the process depicted in Figure 1. 

**Figure 2:**
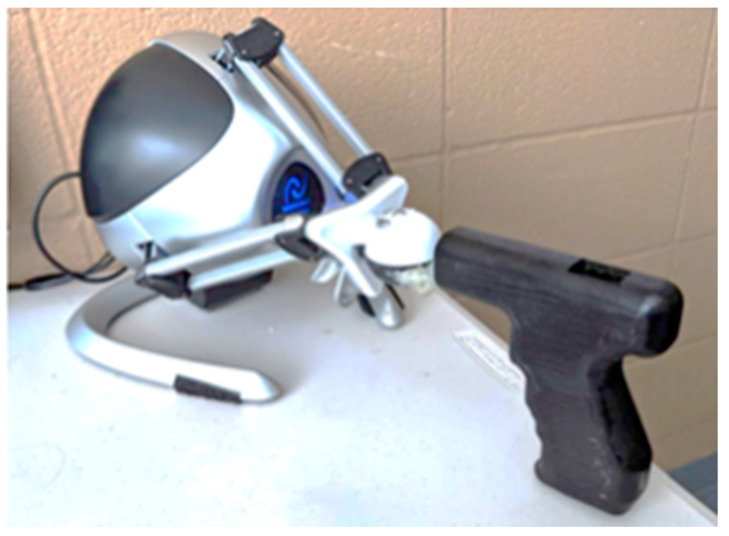
Novint Falcon device covered by a 3D printed drill shape for drilling simulation (similar figure appears in Grant, 2019).

Participants were seated on a chair in front of a desk, facing a 23-inch display monitor. The distance measured from the participants’ eyes to the desktop monitor, on average, was 71.9 cm (±4.8 cm). Speakers were set up directly below the display, to ensure visual and auditory stimuli were coming from a similar direction and so that participants would keep their head directed toward the computer monitor. The drilling simulation was created using the Unity game engine, developed by Unity Technologies (San Francisco, CA, USA), and the Novint Falcon, a low-fidelity haptic device by Novint Technologies (Albuquerque, NM, USA). The drilling simulation was implemented using the Novint Falcon Unity Plugin in addition to the design and 3D printing of a mock drill handle (Figure 2). This was used as a haptic controller and provided force feedback in the haptic trials. The Falcon provides up to 9.0 N of force-feedback, with a resolution of 400 dpi (dots-per-inch).

Participants were able to operate the Falcon device by holding the 3D printed drill handle shape, in place of a real drill. Audio recordings, recorded using a sound booth while drilling through wood, were played throughout the simulation and indicated when the drill initially contacted the wood. The task was to drill two cm into a block of wood. When participants considered they had drilled two cm, they pulled their arm back to the starting position.

Prior to the start of the experiment, participants completed a familiarization phase, performing multiple trials of both haptic and non-haptic conditions. During the familiarization phase, participants were instructed to complete as many trials as needed until they felt confident in their ability to perform the task. This was approximately 20 trials with a range of 16 to 20. After completing the familiarization phase, each participant completed a total of 200 trials pseudo-randomized into blocks of 50 trials per block for a total of 100 haptic and 100 non-haptic trials. There were five-minute breaks in between each block of 50 trials. In both conditions, auditory stimuli were consistent, while force feedback was only provided during the haptic condition [34].

Next, 64-electrode continuous EEG data was recorded using a Waveguard™ 64-electrode EEG cap (ANT Neuro, The Netherlands) during all trials of the simulation, including those with and without haptic feedback. The EEG data were collected using an eego™ mylab amplifier (ANT Neuro, The Netherlands) and a sampling frequency of 1024 Hz. Electrodes were filled with conductive gel and impedance was ≤10 kΩ for each electrode. Haptic and non-haptic trials of the simulation were triggered and coded as events in the EEG data. The trials were presented in four blocks that consisted of 50 trials each. A total of 200 trials, 100 haptic and 100 non-haptic, were coded into each EEG data set. The event trials were presented in a randomized order, where half of the events included haptic feedback. The eego™ mylab amplifier (ANT Neuro, The Netherlands) received the external event triggers, which were programmed in Unity and were relative to the interaction with the Novint Falcon device. 

Initially, all blocks of EEG data were grouped according to their class (haptic or non-haptic). Channel selection and the extraction of the different frequency bands were performed based on previous literature [34,35]. Subsequently, a time, frequency, and nonlinear time-domain feature extraction was performed. After this, a feature selection stage was performed, using two proposals independently, the first one used the maximum relevance minimum redundancy method (MRMR) [36], and the second one complemented MRMR with a statistical independence test. Following this, the classification stage was performed using the feature sets generated by each feature selection proposal. Since the results obtained showed that the feature selection methods were functional and allowed successful classification, it was possible to characterize the behavior of the EEG signals (Figure 1).

### 2.3. Pre-Processing

Initially, blocks of trials were extracted from the EEG recordings using an activation signal generated during the experiment, and each trial was labeled based on feedback (haptic and non-haptic). These blocks of trials were organized into two general groups: haptic and non-haptic feedback. In previous research, nine electrodes were found to be relevant to the analysis of event-related desynchronization in sensorimotor paradigms [34,35]; left frontal (F3), midline frontal (Fz), right frontal (F4), left central (C3), midline central (Cz), right central (C4), left parietal (P3), midline parietal (Pz), and right parietal (P4). 

We also analyzed four different frequency bands: Low Alpha (8–9.5 Hz), High Alpha (10–13 Hz), Low Beta (13–21 Hz), and High Beta (21–30 Hz), as the activation of these bands is related to the response to various tasks, and may signify attentional processes and general task demands [34,37]. Different types of features were extracted based on previous work focused on EEG feature extraction [38,39]. Table 1 indicates the name and type of the 10 base features that were used to train each machine learning model. The limiting values for the Hurst exponent were between 0 and 1 [40], the permutation entropy had values between 0 and *log(3!)* in order to capture the local relationships in the signal [41], and the Higuchi fractal dimension was between 1 and 2 [42].

### 2.4. Feature Selection

Two different groups of relevant features were made in relation to the technique applied. The first technique was the MRMR method. The MRMR method uses the relationship between the F-statistic calculated for each of the extracted features with the target variable or label, and the Pearson correlation applied to each individual feature with the rest of the features in the set. Thus, the higher the score obtained, the greater the relevance of a characteristic [36]. The second method was the MRMR method combined with the Mann–Whitney U statistical test [43]. In the case of the second method, the MRMR was applied first, followed by the Mann–Whitney U test to select the relevant group from the features with the lowest *p*-value (i.e., the features showing the greatest statistical difference).

### 2.5. Classification

Seven classification models were implemented: stochastic gradient descent (SGD), support vector classifier (SVC), decision tree, Naive Bayes, KNN, random forest and an MLP. It should be recalled that there were two data sets, according to each selection method. To avoid possible biases or lack of generalizability in the models trained, a shuffle function was used to mix all samples and, thus, obtain a set of independent samples, rather than a sequence of samples associated with a specific participant. Next, a random group of samples for training the model corresponding to 80% of the total samples was generated. The remaining 20% of the total samples were then used for testing the model. The fundamental objective of this practice is to allow the model to be trained with a sufficiently representative amount of the total population, and then, to test the model using data with which it was never trained, thus avoiding possible biases in the results. Subsequently, each of the models was trained using a heuristic method in which a grid of hyperparameters was generated and combined to train and test each of the models iteratively until the best combination of hyperparameters was obtained (see Table A1 and Table A2 in Appendix A).

### 2.6. EEG Characterization

The classification results obtained allowed the specific analysis of the 10 most relevant features, and consequently, of the channels and frequency bands that provide the most information in the EEG when performing tasks related to haptic feedback.

## 3. Results

### 3.1. EEG Characterization

Ten features were extracted from nine EEG channels and four different frequency bands. In total, 360 features were obtained.

### 3.2. Feature Selection

**Figure 3:**
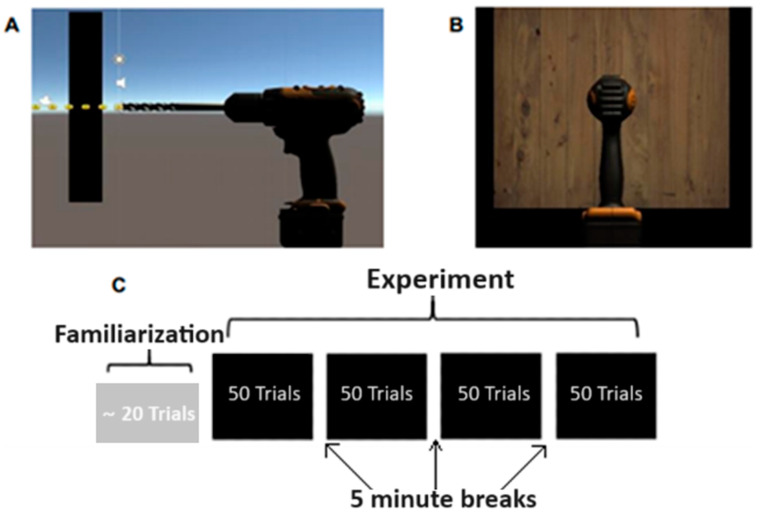
Simulation scenes and experimental protocol for data collection. (**A**) Side view was only observable during the familiarization trials. (**B**) The front view was displayed during all experimental trials. (**C**) Experimental design. (Panels **A** and **B** are screen shots of drill with similar figures in https://www.mdpi.com/2076-3425/10/1/21, accessed on 20 August 2024).

In order to determine the relative weighting of each feature to the model, the 20 most relevant features were determined using MRMR (Figure 3). This method calculates a score that can be interpreted as the quotient between the maximum relevance that a feature has with respect to the target variable and the minimum redundancy with respect to the other features. That means that the higher the score, the more relevant the feature. The top 20 most relevant features confirmed the results of the first dataset for the machine learning approach.

The second dataset was generated by applying the Mann–Whitney U test to the first dataset. The reason for this is because the scores from the MRMR method do not provide information related to the difference between the classes and, therefore, statistical tests could be a solution to improve the performance of the models that use features selected using MRMR for training. Table 2 shows the *p*-values for the 14 features that showed statistical differences between the classes (*p* < 0.05).

### 3.3. Classification

Classification of both data sets was performed using different batches. The first classification used as an input for training the models using the 5, 10, 15, and 20 most relevant features according to the relevance score yielded by MRMR and the total set of extracted features (Table 3). Regarding the second data set, the 5, 10, and 14 features that presented a more marked statistical difference were used to train the models (Table 4).

### 3.4. EEG Characterization

According to the classification results, the 10 most relevant features correspond to the first 10 features in Table 2. In this sense, topographic maps were generated in order to visualize the behavior of all channels with respect to the numerical value of the features and frequency bands that provide the most information. It is important to clarify that the intensity of the color scale in the maps does not represent brain activity directly but shows the average value of a channel for a specific feature in a frequency band as indicated by the scales that are included with each map.

**Figure 4:**
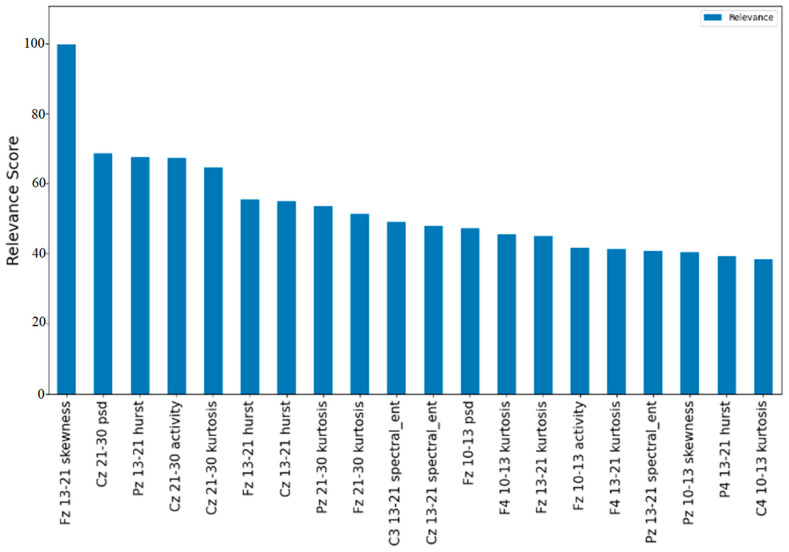
Score of the top 20 most relevant features according to MRMR. Maximum score of 100.

**Figure 5:**
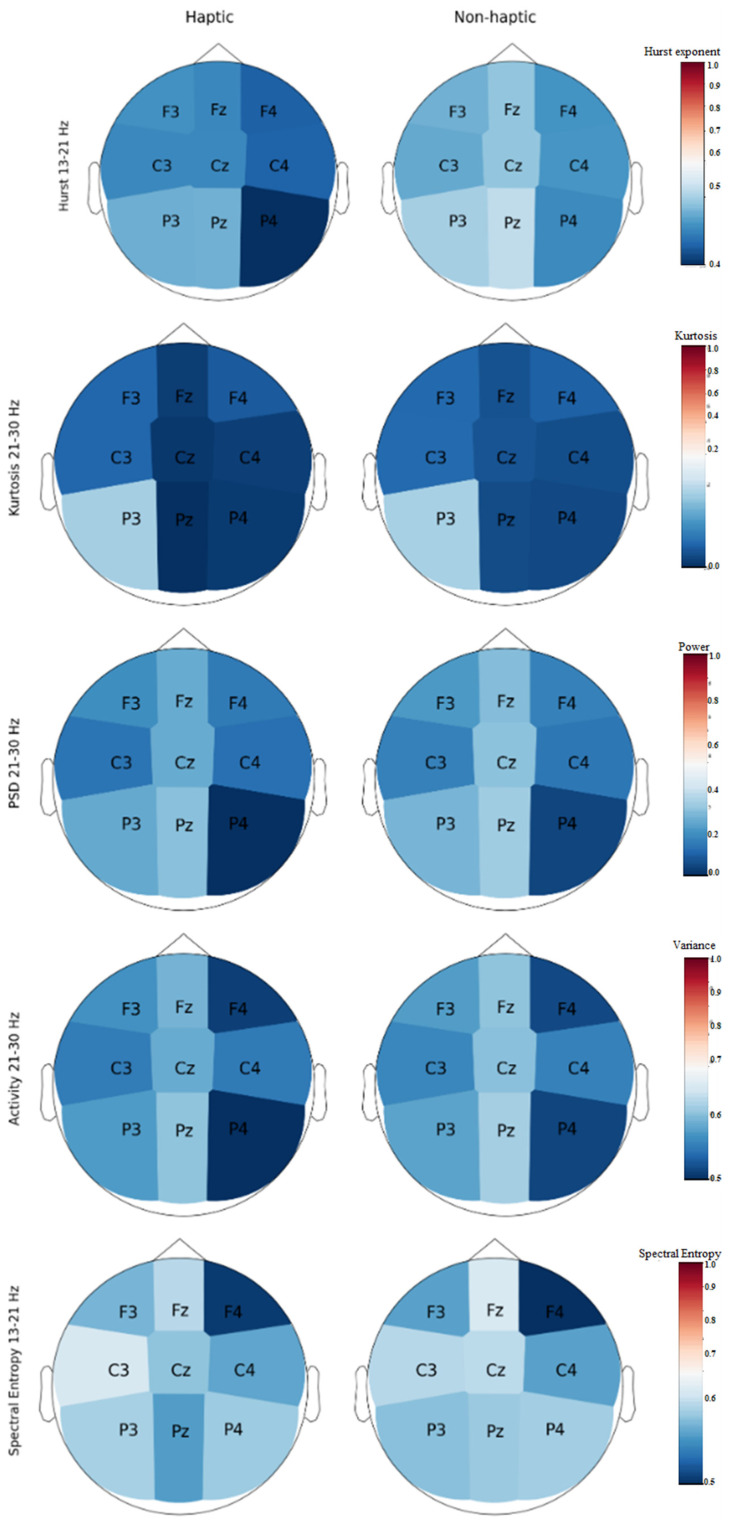
Topographic map of the most relevant features and frequency bands. Colors close to red represent high mean values (close to 1), and colors close to dark blue represent low mean values (close to 0).

Figure 4 demonstrated the difference between the mean values of the best features in the most relevant frequency bands for each class (haptic and non-haptic feedback processing). Before generating the topographic maps, the values were normalized between 0 and 1. In order to make it easier to visualize the differences in the figure, each pair of topographic maps has its own color scale (Figure 5). In general, the channels with the most relevant information were Cz, Pz, Fz, P4, and C3. The Hurst exponent in the 13–21 Hz frequency band had the midline and P4 as relevant channels. The kurtosis had two relevant frequency bands, low beta and high beta, and for each, Cz and F4, respectively, were the relevant channels. The PSD and variance (activity according to Hjorth parameters) had the same frequency bands and relevant channels: Fz for high alpha and Cz for low beta. Finally, spectral entropy for low beta had C3, Cz, and Pz as relevant channels.

## 4. Discussion

To our knowledge, this research is the first to utilize the current machine learning approaches to identify neural correlates associated with simulations completed in the presence of haptic feedback. Given the increasing prominence of high-fidelity simulation in rehabilitation and the impact of this approach, this can enhance the understanding of ways to optimize the design and implementation of treatments. For instance, the ability to accurately differentiate neural processing under varying sensory conditions (e.g., haptic vs. non-haptic) could transform rehabilitation approaches that rely on simulation for specific populations. Machine learning features could also be used to determine if the addition of haptic feedback improves neural processing and rehabilitation outcomes. One such population is those with dementia, for whom current research aims to identify optimal methods for the implementation of VR-supported therapy [44]. 

The current features used proved useful for classifying haptic feedback processing and non-haptic feedback processing from EEG data. The 10 most relevant features include hurst exponent, kurtosis, power spectral density, activity, spectral entropy, and skewness. These top 10 were the combination of all feature types included in this study (i.e., all these features provide time domain, frequency domain, and nonlinear information), so we recommend the use of these as they can provide different types of valuable information. In addition, the importance of feature selection techniques in machine learning applications should be highlighted. Table 3 shows the usefulness of MRMR, as, with only 20 features, it was possible to obtain accuracy percentages almost equal to those obtained using all of the features and it was observed that in the worst case for the best classifiers, the reduction in the accuracy percentage is only 10%. Similarly, the use of the Mann–Whitney U test was complementary to MRMR, since this allowed a reduction to 10 features and to rank them according to their *p*-value (see Table 2). Table 4 shows that the most relevant features can be used to generate models with good performance and less processing time, because with only 10 (representing only 2.77% of the total), it was possible to obtain accuracy percentages of 99% using KNN, RF, and MLP, and 98% for SVC and DT. The second feature selection method is a novel finding, and we are not aware of any previous work that has used the same technique to classify EEG signals collected with and without haptic feedback. For differentiating haptic vs. non-haptic trials, the 10 most relevant features closely represent the optimal number required, given that the performance accuracy at 99% was as good as using all 360 features. Even using only five features as inputs, regardless of the set used, gave high accuracy, suggesting that that these features have the capacity to distinguish between neural processing of haptic vs. non-haptic feedback.

The generated models yielded promising results in addition to surpassing the results of previous work [26,27,28,29,30,31,32,33]. It was also demonstrated that these models can be effective tools to identify EEG signals associated with haptic feedback. Some of the previous work aimed to classify EEG signals collected during different tasks in VR. Therefore, they used complex methods such as neural networks [26] and CNN [28], obtaining accuracy percentages of around 96%. When compared to classical machine learning methods, whose results, despite being inferior to those obtained with deep learning methods, are equally promising, yielding 86% [26] and 97% accuracy using KNN [33]. The work that classified EEG signals according to the haptic feedback received by the participants, also used from CNN, obtaining 84.56%, 93.96%, and 95.89% accuracy when using different numbers of electrodes [31], to machine learning classifiers such as SVM [29,32] or XGBoost [30], where the best results obtained were 85.46% and 85%, respectively.

Regarding the behavior of the mean values of the relevant features described in Table 3, these showed higher mean values for non-haptic feedback processing, except the C3 channel for spectral entropy at low beta. In terms of the EEG signal, this means that non-haptic feedback processing demonstrates higher variance, stability, and power in the frequency bands associated with mental activity. This may be because drilling is a task typically associated with haptic feedback, and in its absence, the individual required differed neural processing to complete the task.

Previous research has shown that in this context, haptic feedback can contribute to a better performance of the task [34]. As a result, this paper not only presents a classification exercise using machine learning approaches, but also a general characterization of the information provided by the analyzed dataset. This was achieved using an approach that assessed the channels, frequency bands, and most relevant features to the interpretation of their behavior. Therefore, this can be considered as a valuable contribution concerning the impact of haptic feedback on VR tasks and EEG signals.

On the other hand, these results suggest that machine learning models may represent valuable tools not only in rehabilitation applications, allowing for better design of rehabilitation interventions and possible early diagnosis, but also in prevention and diagnosis. The information provided by the relevant features could allow early identification of anomalies in neural processing behavior associated with haptic processing. This would represent an advantage not only for the patient, by avoiding the progression of neuromotor pathologies, but also for the health care system. Likewise, the use of these characteristics allows the development of less complex models that yield promising results, thus allowing a decrease in the processing time and in the use of computational resources compared to other more complex techniques such as CNN, for example.

Limitations to note include the machine learning approach being applied to a relatively small data set (15 participants), allowing only the training and validation stages. Future work should aim to use a larger data set with an increased number of participants, which may allow the implementation of a validation system confirmed by training, validation, and test groups. Future work could also investigate the impact of visual and auditory immersion as well as different types of haptic tools to determine if the machine learning approach can be generalized to other cases. Additionally, differences in neural activation identified by machine learning could be compared to task performance with and without haptic feedback.

## 5. Conclusions

This study presents a machine learning approach that aimed to classify EEG signals representative of neural circuits associated with haptic and non-haptic sensory feedback when completing a simulated drilling task. The results obtained indicate that machine learning applied to EEG is able to discriminate brain activity when performing a task with and without haptic feedback. The generated models allowed us to obtain accuracy percentages of 99% when using the 10 most relevant features (representing only 2.77% of the total number of features), thus outperforming those of previous related research, and likewise, optimizing variables such as processing time and the use of computational resources, compared to using a large number of features or using more complex classifiers such as CNN. The results improve our understanding of the neural differences associated with haptic feedback. These machine learning EEG markers allow us to compare neural processing differences when there are haptic differences in user interfaces and to determine whether these neural differences predict the transfer of learning from VR training to real-world tasks. In this sense, haptic feedback is becoming increasingly important in creating high-fidelity simulations and virtual reality environments for rehabilitation applications.

## Figures and Tables

**Table 1:** Types of features extracted.

Types of Features	Features
	Activity (Variance)
Time Domain	Mobility
	Complexity
Frequency Domain	Power Spectral Density (PSD)
Entropy	Permutation
	Spectral
Non-Linear	Higuchi’s Fractal Dimension
	Hurst’s Exponent
Statistical	Skewness
	Kurtosis

**Table 2:** *p*-values for the 14 features of the first dataset that show statistical differences between the classes (*p* < 0.05). The name of the feature is given as follows: Channel_Frequency Band_Feature.

Feature Name	*p*-Value
Pz 13–21 Hz Hurst	1.20 × 10^8^
Fz 13–21 Hz Hurst	1.72 × 10^8^
Cz 13–21 Hz Hurst	4.65 × 10^8^
Cz 21–30 Hz Kurtosis	8.98 × 10^6^
Cz 21–30 Hz PSD	7.69 × 10^5^
Cz 21–30 Hz Activity	8.20 × 10^5^
P4 13–21 Hz Hurst	2.34 × 10^4^
C3 13–21 Hz Spectral_Ent	7.78 × 10^4^
Cz 13–21 Hz Spectral_Ent	1.83 × 10^3^
Pz 13–21 Hz Spectral_Ent	1.07 × 10^2^
Pz 10–13 Hz Skewness	1.29 × 10^2^
F4 13–21 Hz Kurtosis	2.82 × 10^2^
Fz 10–13 Hz Activity	2.89 × 10^2^

**Table 3:** Percentage of accuracy for each classifier using the first data set as inputs.

	5 Best Features	10 Best Features	15 Best Features	20 Best Features	All Features
SGD	62	65	66	73	93
SVC	88	91	92	99	100
DT	85	86	92	96	100
GNB	54	59	52	66	58
KNN	90	92	94	99.4	100
RF	88	90	95	99.7	100
MLP	90	91	94	99.2	100

**Table 4:** Percentage of accuracy for each classifier using the second data set as inputs.

	5 Best Features	10 Best Features	14 Best Features
SGD	66	71	71
SVC	84	98	99
DT	88	98	96
GNB	64	65	67
KNN	90	99	99
RF	90	99	100
MLP	91	99	99

## Data Availability

The de-identified EEG data will be made available upon reasonable request due to the requirements of our Research Ethics Board.

## Appendix A

**Table A1:** Combination of hyperparameters to obtain the models whose results are presented in Table 3.

	5 Best Features	10 Best Features	15 Best Features	20 Best Features	All Features
SGD	max_iter = 10 tol = 0.0001	alpha = 0.01 max_iter = 100 tol = 0.0001	alpha = 0.01 max_iter = 100 tol = 0.0001	alpha = 0.01 max_iter = 100 penalty = ‘l1’ tol = 0.0001	max_iter = 100 penalty = ‘l1’ tol = 0.0001
SVC	C = 100 tol = 0.01	C = 100 tol = 0.01	C = 100 tol = 0.01	C = 10 kernel = ‘poly’ tol = 0.01	C = 100 kernel = ‘poly’ tol = 0.01
DT	alpha = 0.001	alpha = 0.001 criterion = ‘entropy’ splitter = ‘random’	alpha = 0.01 criterion = ‘entropy’	alpha = 0.01 splitter = ‘random’	alpha = 0.01 splitter = ‘random’
GNB	var_smoothing = 0.1	var_smoothing = 100	var_smoothing = 0.0001	var_smoothing = 10	var_smoothing = 100
KNN	leaf_size = 10 metric = ‘cityblock’ n_neighbors = 3	leaf_size = 10 metric = ‘cityblock’ n_neighbors = 7 weights = ‘distance’	leaf_size = 10 metric = ‘cityblock’ n_neighbors = 7	leaf_size = 10 metric = ‘cityblock’ n_neighbors = 1	leaf_size = 10 metric = ‘cityblock’ n_neighbors = 19
RF	max_depth = 10 n_estimators = 50	max_depth = 5 n_estimators = 50	max_depth = 10 n_estimators = 50	criterion = ‘entropy’ max_depth = 10 n_estimators = 50	max_depth = 5 n_estimators = 10
MLP	alpha = 0.001 hidden_layer_sizes = 100 max_iter = 5000 solver = ‘lbfgs’	activation = ‘tanh’ alpha = 0.001 hidden_layer_sizes = 50 max_iter = 5000	activation = ‘logistic’ alpha = 0.001 hidden_layer_sizes = 100 max_iter = 5000	activation = ‘logistic’ alpha = 0.001 hidden_layer_sizes = 50 max_iter = 5000	alpha = 0.001 hidden_layer_sizes = 50 max_iter = 5000

**Table A2:** Combination of hyperparameters to obtain the models whose results are presented in Table 4.

	5 Best Features	10 Best Features	14 Best Features
SGD	loss = ‘perceptron’ max_iter = 100	loss = ‘huber’ max_iter = 100 tol = 0.0001	alpha = 0.01 loss = ‘log’ max_iter = 10 tol = 0.01
SVC	C = 1 tol = 0.01	C = 1 tol = 0.01	C = 100 tol = 0.01
DT	ccp_alpha = 0.001 max_features = ‘auto’ splitter = random	ccp_alpha = 0.001 max_features = ‘auto’	ccp_alpha = 0.001 max_features = ‘auto’
GNB	var_smoothing = 10	var_smoothing = 1	var_smoothing = 1
KNN	leaf_size = 10 metric = ‘euclidean’ n_neighbors = 11 weights = ‘distance’	leaf_size = 10 metric = ‘cityblock’ n_neighbors = 1	leaf_size = 10 metric = ‘cityblock’ n_neighbors = 1
RF	max_depth = 10 max_features = ‘auto’ n_estimators = 50	max_depth = 10 max_features = ‘auto’ n_estimators = 200	criterion = ‘entropy’ max_depth = 10 max_features = ‘auto’ n_estimators = 500,
MLP	activation = ‘tanh’ alpha = 0.001 hidden_layer_sizes = 20 max_iter = 5000 solver = ‘lbfgs’	activation = ‘logistic’ alpha = 0.001 hidden_layer_sizes = 50 max_iter = 5000 solver = ‘lbfgs’	alpha = 0.001 hidden_layer_sizes = 50 max_iter = 5000
